# Influence of online opinions and interactions on the Covid-19 vaccination in Chile

**DOI:** 10.1038/s41598-022-23738-0

**Published:** 2022-12-09

**Authors:** Claudio Villegas, Abril Ortiz, Víctor Arriagada, Sofía Ortega, Juan Walker, Eduardo Arriagada, Alexis M. Kalergis, Cristián Huepe

**Affiliations:** 1 Social Listening Lab SoL-UC, Pontificia Universidad Católica de Chile, Santiago, Chile; 2 School of Anthropology, Universidad Academia de Humanismo Cristiano, Santiago, Chile; 3 School of Communications, Pontificia Universidad Católica de Chile, Santiago, Chile; 4 Departamento de Genética Molecular y Microbiología, Facultad de Ciencias Biológicas, Millennium Institute on Immunology and Immunotherapy, Pontificia Universidad Católica de Chile, Santiago, Chile; 5 Departamento de Endocrinología, Facultad de Medicina, Pontificia Universidad Católica de Chile, Santiago, Chile; 6 CHuepe Labs, Chicago, IL 60622 USA; 7 Northwestern Institute on Complex Systems and ESAM, Northwestern University, Evanston, IL 60208 USA

**Keywords:** Epidemiology, Mathematics and computing

## Abstract

We analyze 6 months of Twitter conversations related to the Chilean Covid-19 vaccination process, in order to understand the online forces that argue for or against it and suggest effective digital communication strategies. Using AI, we classify accounts into four categories that emerge from the data as a result of the type of language used. This classification naturally distinguishes pro- and anti-vaccine activists from moderates that promote or inhibit vaccination in discussions, which also play a key role that should be addressed by public policies. We find that all categories display relatively constant opinions, but that the number of tweeting accounts grows in each category during controversial periods. We also find that accounts disfavoring vaccination tend to appear in the periphery of the interaction network, which is consistent with Chile’s high immunization levels. However, these are more active in addressing those favoring vaccination than vice-versa, revealing a potential communication problem even in a society where the antivaccine movement has no central role. Our results highlight the importance of social network analysis to understand public discussions and suggest online interventions that can help achieve successful immunization campaigns.

## Introduction

The current global Covid-19 pandemic has demonstrated the importance of mass vaccination campaigns and the need to promote vaccine acceptance to maximize immunization coverage. However, it has also shown that these efforts can be strongly hindered by vaccine skepticism and misinformation propagated online through social media, especially in polarized societies^1^.

Although each country has faced the pandemic differently^2^, the Chilean experience can provide a unique case study of online conversations in a society with widespread internet access^3^ that has had high infection rates^4–6^ and a large percentage of its population vaccinated within a short time span^7–9^, all during a period of considerable sociopolitical conflict^10–12^. Indeed, in less than 2 years, Chile went from facing one of the highest transmission rates in the world, in June 2020^4–6^, to reaching the first place in Bloomberg’s Covid Resilience Ranking, in December 2021^13^, after a successful vaccination campaign. Since the first case of SarsCov2 was detected on March 3rd, 2020^14^, Chile, like many other countries, has experienced widely different levels of transmission, hospitalizations, and mortality^7^. Still, unlike most other countries, Chile implemented a highly effective immunization campaign, fully vaccinating over 90% of its target population by October 2021^6,8,9^. Despite this success, the country has a large population that resist vaccination; more than 1.1 million had not yet received a single dose or completed their vaccination scheme by January 2022^4^. It is important to note that Covid-19 vaccination is completely voluntary in Chile, although it is strongly encouraged by the authorities with tools such as a “*mobility pass*”^15^, which can only be obtained after full immunization and has been required in most public spaces and larger social gatherings.

The Chilean experience is also a valuable case study for analyzing the relationship between online views and vaccination because the pandemic developed shortly after the eruption of a period of considerable sociopolitical conflict that used social networks for rallying and organizing, which resulted in highly polarized online discussions and communities^16^. Indeed, on October 18, 2019, just 5 months before Covid reached Chile, the country experienced its biggest social upheaval in 30 years^10^. A series of massive peaceful protests and acts of civil disobedience demanding more social justice and socioeconomic guarantees became what is referred to as a “*social outburst*” (“*estallido social*”) that questioned established authorities and institutions^11^. These mobilizations also led to violent crashes, looting, and vandalism that damaged public and private property^10–12^. Although much of the turmoil had subsided when the pandemic reached Chile, during the summer vacation period, mass protests were expected to return^12^ at the exact time that civil liberties were restricted as part of a series of public health measures. This further polarized online discussions regarding the pandemic response^11^.

Notwithstanding these underlying sociopolitical conflicts, the Chilean government, opposition, and civil society broadly accepted the need for strong Covid-19 mitigation measures^4,8,9,17^. Most people adhered to protective behaviors, such as social distancing or the use of masks^8,9^, and accepted substantial restrictions on the freedom of movement and assembly^15^. In February 2021, Chile started a free mass vaccination campaign, based mainly on the CoronaVac vaccine from Sinovac^18–21^, which was successfully distributed through an efficient primary health care system and hospital network^9^. By May 2021, Chile had implemented its *mobility pass*, required to avoid restrictions in most social activities^4^. In August, the booster shot campaign started^22^ and, in September, the vaccination of children over six^23^. Although these measures received widespread acceptance, anti-vaccine communities still have a significant presence in Chilean social networks, with polls showing at the beginning of the pandemic that the Chilean anti-vaccine and vaccine reticent population mirrored the relatively elevated rates found in other countries^24^. (See supplementary text for an overview of Chile’s sociopolitical and sociosanitary context during the period of our study.)

The context described above shows that Chilean online conversations related to the Covid-19 vaccination process can be expected to contain a rich diversity of positions that represent well the views and discussions that are currently happening, or will soon develop, in many other countries. The quantitative and qualitative analyses in this paper can therefore help evaluate and understand the properties and interactions of pro- and anti-vaccination communities, both in general and in societies that can achieve high vaccination rates, which could in turn help design online communication strategies and interventions that result in higher vaccination rates around the world.

## Data analysis and account classification

In this study, we analyzed all Chilean tweets related to the Covid-19 vaccines and vaccination process produced during a period of 6 months, scoring them with a machine learning algorithm in a range from the most pro-vaccine to the most anti-vaccine. We will show below that the resulting distribution of scores and content analysis leads to four naturally emerging categories, each with distinct characteristics regarding their tweeting practices and interaction networks.

We began by gathering 351,573 tweets (generated by 59,252 different accounts) related to the vaccines or the vaccination process, produced in Chile from March 1st until August 31st, 2021. During this period, the fraction of the Chilean population that received at least one dose of the vaccine went from 18% to over 80%, while the number of confirmed Covid infections and deaths almost doubled, increasing from 43,189 to 85,316 per million and from 1075 to 1923 per million, respectively^4^. The high vaccination rates and number of casualties makes this an ideal period for sampling a broad range of conversations. Note that we analyze Twitter because it is the only major digital social network in which the content, origin, and destination of all interactions on a given subject can be accessed without any privacy concerns.

We created a training set by randomly selecting 185 accounts from all those with 10 to 500 tweets in the dataset and manually classifying them as favoring or disfavoring vaccination, based on the expected effect of their content on readers. Note that accounts classified as disfavoring vaccination do not only include anti-vaccine accounts, but also those that expressed skepticism regarding the vaccine effectiveness or the vaccination process. On the other hand, accounts classified as favoring vaccination express either explicitly or implicitly pro-vaccine views. We thus identified 115 accounts favoring vaccination and 70 accounts disfavoring vaccination, which generated a total of 6331 and 6442 tweets, respectively. These sampled a relatively even distribution of the language used by a range of positions regarding vaccination, from the most pro-vaccine to the most anti-vaccine. (See Methods for further details on the data collection, manual classification procedure, and resulting categories.)

After completing the classification process, all tweets generated by the classified accounts were used as the training set for a TensorFlow machine learning algorithm implemented in the Keras R package^25^, by labeling them with a training score of 0 or 1 when originating from an account respectively favoring or disfavoring vaccination. The resulting model thus provides a pro/anti-vaccine score to each tweet that we then average over all tweets from each account, to place it in the 0 (pro-vaccine) to 1 (anti-vaccine) account type spectrum. This approach produced 85% accuracy and a loss of 44% in reproducing ground truth (manually classified) accounts as favoring or disfavoring vaccination. Finally, using this model, we computed the pro/anti-vaccine score for all the accounts in our dataset, including those in the training set.

## Results

### Account categories, properties, and activity

**Figure 1:**
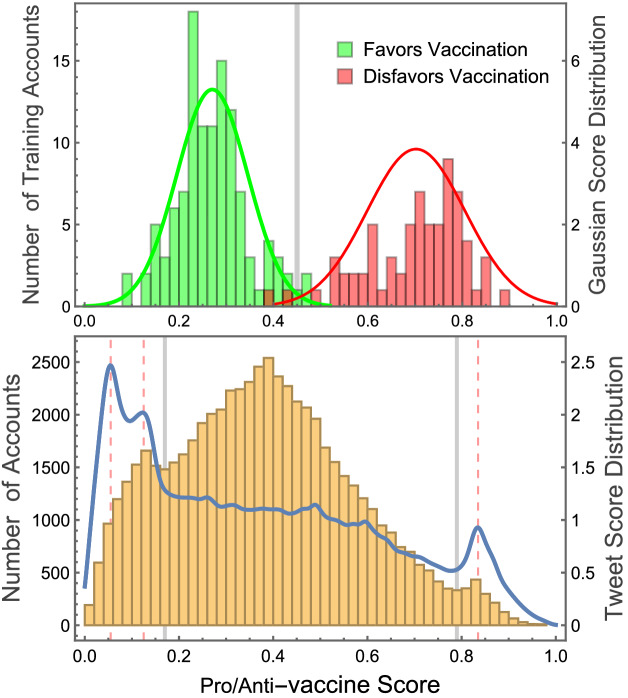
Score distributions and definition of account categories (pro-vaccine, promoter, inhibitor, anti-vaccine). **Top:** Histograms of pro/anti-vaccine scores of the two training sets (one favoring and one disfavoring vaccination), and corresponding Gaussian distributions, used to estimate the boundary between accounts that favor or disfavor vaccination (vertical gray line). **Bottom:** Histogram of the scores of all accounts (orange bins of width 0.02) and distribution of individual tweet scores (blue curve). The histogram maximum corresponds to the score of the most common views. The local maxima at both sides and peaks in the distribution curve (labeled by red dashed lines) match the scores of activist messages, since these use repetitive language that results in similar values. The histogram’s local minima are thus used to define the boundaries between pro/anti-vaccine activist and moderate accounts (vertical gray lines).

Figure 1 presents the pro/anti-vaccine score distributions of the accounts and tweets gathered in our analyses, which we will use to define four categories of positions on Covid-19 vaccination: pro-vaccine, vaccine promoter, vaccine inhibitor, and anti-vaccine. The top panel displays histograms of the scores of the training set accounts that favor and disfavor vaccination, with their corresponding Gaussian distributions (with the same mean and standard deviation values). The two training sets split into well-differentiated communities of either low (favoring vaccination) or high (disfavoring vaccination) scores. From these distributions, we can define an approximate boundary at score 0.45 (estimated from the figure) so that accounts with scores below this threshold are defined as pro-vaccine or promoters, and above it, as anti-vaccine or inhibitors. The bottom panel of Fig. 1 presents a histogram of the scores of all accounts (orange bars), displaying three local maxima. The central one can be easily explained, as it corresponds to the typical score of mainstream users. The two lower local maxima, near both edges of the histogram, are more interesting; they appear at scores that are typical of the monolithic language used by pro- or anti-vaccine activists. Indeed, when plotting the distribution of scores per individual tweet (blue curve), we find strong peaks near these maxima, which correspond to scores associated to standardized, repetitive messages that characterize activism (see Methods for a description of the types of posts that we find with these scores). We can thus define the local minima between the central and lateral maxima (estimated at scores 0.17 and 0.79) as the boundaries between pro- and anti-vaccine accounts that actively promote their views with monolithic statements and more moderate accounts that discuss their promoting or inhibiting positions through diverse messages.

Using these boundaries, we were able to classify the accounts based not only on whether they favor or disfavor vaccination but also on their level of activism in propagating their viewpoints. We defined all accounts with scores from 0 to 0.17 as pro-vaccine, from 0.17 to 0.45 as promoters, from 0.45 to 0.79 as inhibitors, and from 0.79 to 1 as anti-vaccine. Note that all four categories emerged naturally from our language analysis, despite starting from a binary training set. Although their exact ranges will always be somewhat arbitrary, we verified that small changes do not significantly affect the results presented below.

**Figure 2:**
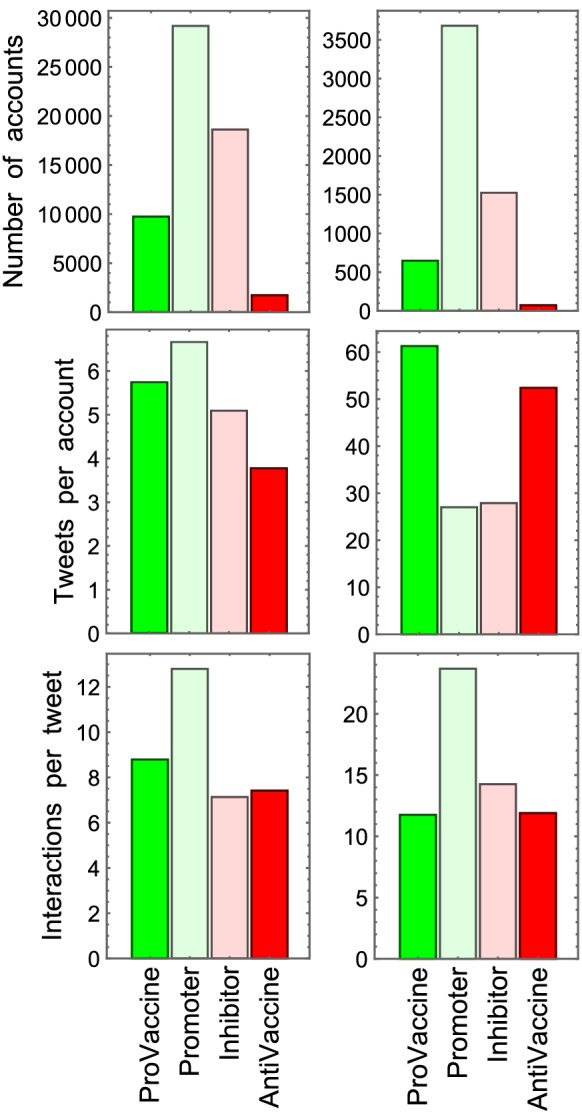
Accounts and activity per category. Bar charts of the number of accounts, mean number of tweets per account, and mean number of interactions per tweet for the four account categories (pro-vaccine, promoter, inhibitor, anti-vaccine), averaged either over all accounts (left column) or over the top 10% with most interactions (right column). Promoter accounts appear as the largest category, followed by inhibitors. The top 10% pro/anti-vaccine activist accounts tweet significantly more than the moderates. Despite their smaller number and lack of centralized organization, anti-vaccine accounts generate significant activity.

Figure 2 compares the activity of the four account categories defined above. Its left column includes all users and its right column, only the top 10% of accounts with the highest total number of interactions. The top panels show that most accounts have moderate (promoter or inhibitor) positions and that anti-vaccine accounts are a relatively small minority. Regarding their activity, the central panels show that the mean number of tweets per account ranges roughly from 4 to 7 in all categories but can be more than 50 in the pro- and anti-vaccine accounts among the 10% with most interactions, which is about double the number of tweets produced by moderate accounts. It is remarkable that individual anti-vaccine activists can reach activity levels similar to those of pro-vaccine accounts, since the latter are often institutional and thus have dedicated communication teams. Finally, regarding the level of success in propagating their views, the lower panels show that pro- and anti-vaccine accounts receive a similar number of interactions, which is also remarkable given Twitter’s pro-vaccine information campaigns and restrictions on the propagation of anti-vaccine messages.

**Figure 3:**
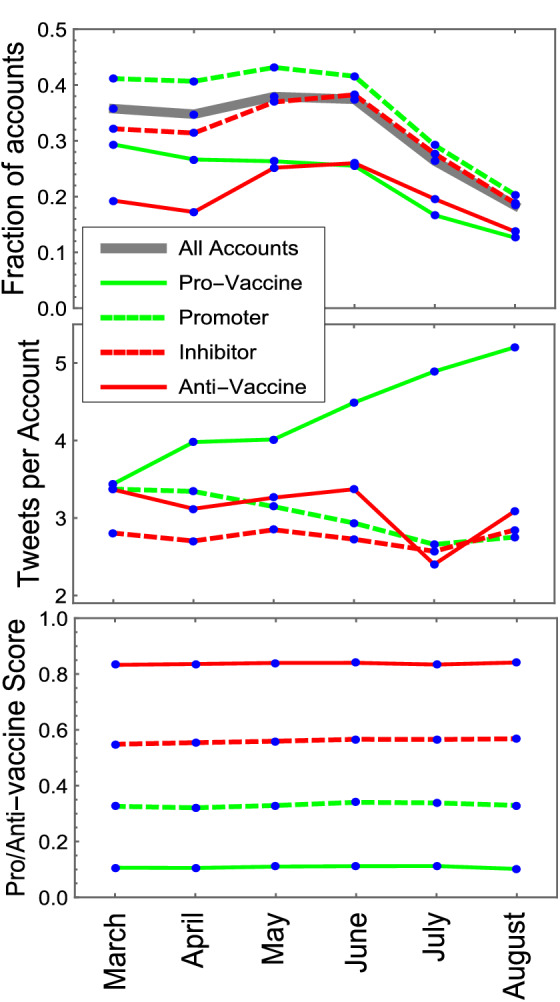
Temporal dynamics of the accounts and activity per category. Monthly values of the fraction of active accounts (that tweeted at least once in a month), number of tweets per account, and opinion scores for each category. Although the number of active accounts appears to increase in periods of controversy regarding vaccination, their mean opinions and activity do not significantly change. The sustained activity increase of pro-vaccine accounts can be attributed to the proliferation of informative messages and appeals to get vaccinated by organizations related to the vaccination campaign.

Figure 3 presents the activity of the different categories over time. The top graph shows the fraction of accounts of each type that were activated per month (i.e., that tweeted at least once), the central graph, their mean number of tweets, and the bottom graph, their mean score. We find that the number of activated accounts per category changes with the contingencies of the vaccination process, but not their typical tweeting behavior or mean opinions. Indeed, in June, Chile experienced a large peak in the number of cases while it surpassed 50% of its population vaccinated with at least one dose, which led to high levels of public controversy regarding the effectiveness of the vaccination process (see supplementary text). During this period, the number of activated accounts increased in all categories, except for the pro-vaccine category, which appears to follow a more institutional approach that does not react to contingencies. In addition, activated anti-vaccine accounts showed the highest relative increase. On the other hand, the mean number of tweets per account and mean score remained relatively constant for each category, seeming not to be strongly affected by the public debate.

### Interaction network and information flow

**Figure 4:**
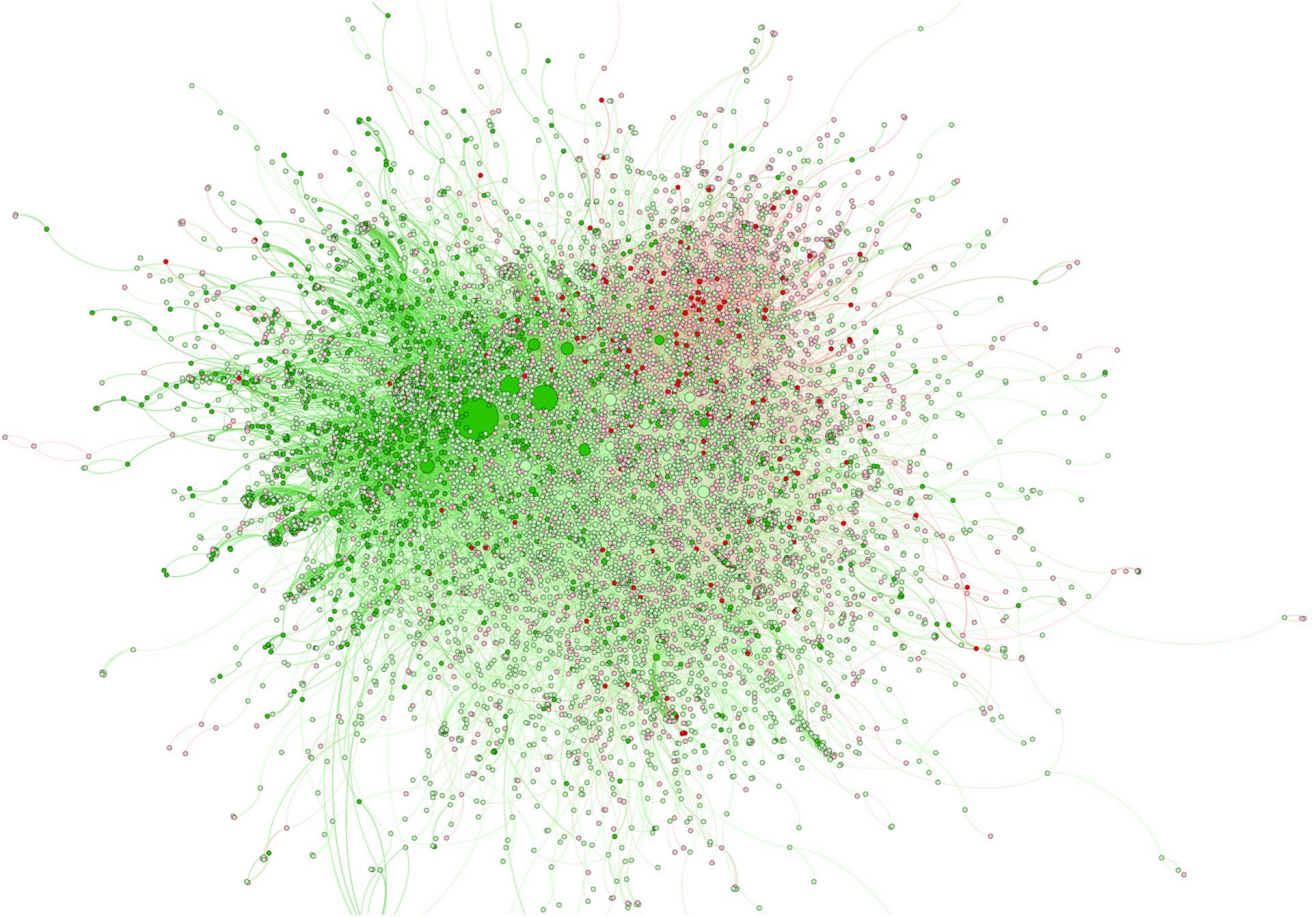
Network of Twitter interactions regarding the Covid-19 vaccination process in Chile. Each node is an account; each link represents replies, citations, or references between accounts. The displayed network is the giant component of all classified nodes that tweeted at least twice about the vaccination process during the 6 months of collected data, consisting of 10,275 nodes and 23,336 links. The anti-vaccine (dark red) and inhibitor accounts (light red) appear to group at the top right side while the pro-vaccine (dark green) and promoter (light green) accounts appear to group at the top left side. This is due to the display algorithm, which places closer together the more highly connected groups of nodes.

We now turn our attention to the network of interactions between accounts. Figure 4 displays the giant component of all accounts that interacted during the period of analysis^26,27^. Each node corresponds to an account, colored according to its category, and the links represent interactions between accounts (replies, citations, or references). We observe that accounts that favor or disfavor vaccination do not segregate into clearly defined communities, although the force-directed graph presented in the figure does tend to place some of the nodes that belong to the same category near each other, evidencing the presence of enhanced interactions between them. The anti-vaccine community thus seems centered in the top-right region, whereas pro-vaccine accounts mainly appear in the top-left region, where we also find several accounts with high PageRank^28,29^ values (represented by large nodes) that are well connected because they typically belong to organizations or public figures involved in vaccine promotion.

**Figure 5:**
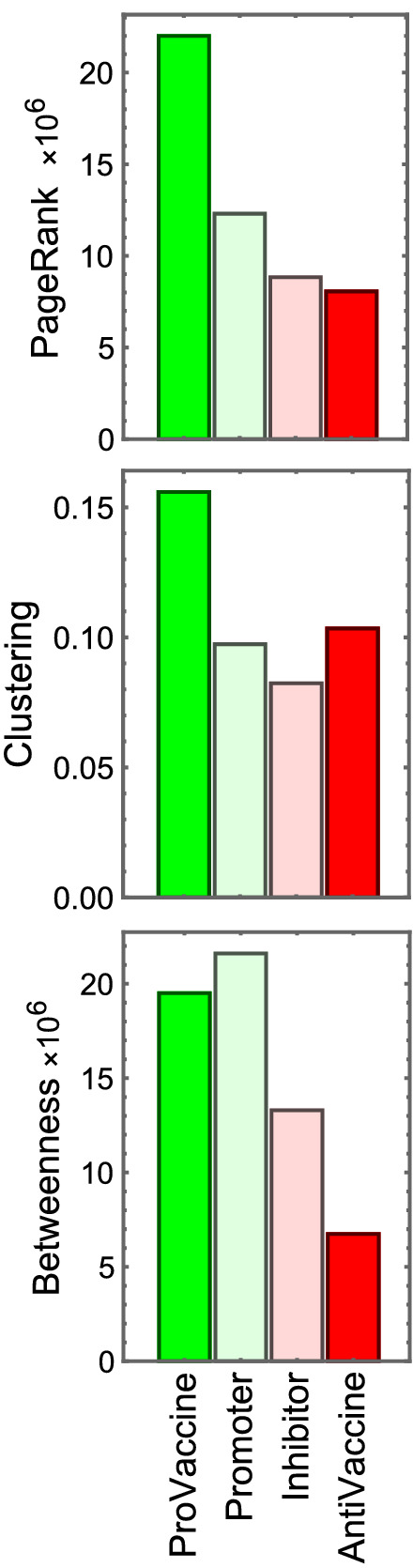
Bar charts displaying three standard network measures, averaged over all nodes in each category, within the complete network (also including non-classified nodes that interacted with the classified accounts). The top two charts show that pro-vaccine accounts have better quality connections (higher PageRank) but tend to form more triangular structures with nodes that also interact with each other (higher clustering coefficient), which is consistent with the overrepresentation of accounts of institutions and authorities in this category. The bottom chart shows the lower betweenness centrality of the inhibitor and anti-vaccine accounts, which can be interpreted as a positive diagnostic since it reflects their peripheral positions in the national conversation.

Figure 5 displays three charts that characterize the mean topological properties^30,31^ of the nodes of each category in the complete interaction network that includes all categories and the unclassified nodes linked to them. The mean PageRank reflects the influential position of pro-vaccine accounts in Chilean society. The clustering coefficient, however, shows that these tend to create tight-knit groups; most of their connections also interact with each other and thus have reduced outreach to other communities. This may result in part from their observed tendency to tag other pro-vaccine accounts. Finally, the low betweenness centrality of anti-vaccine accounts shows that they tend to appear in the periphery of the conversations. This is consistent with the widespread vaccination acceptance in Chile, where anti-vaccine communities have not connected with any mainstream narrative or sociopolitical movement.

**Figure 6:**
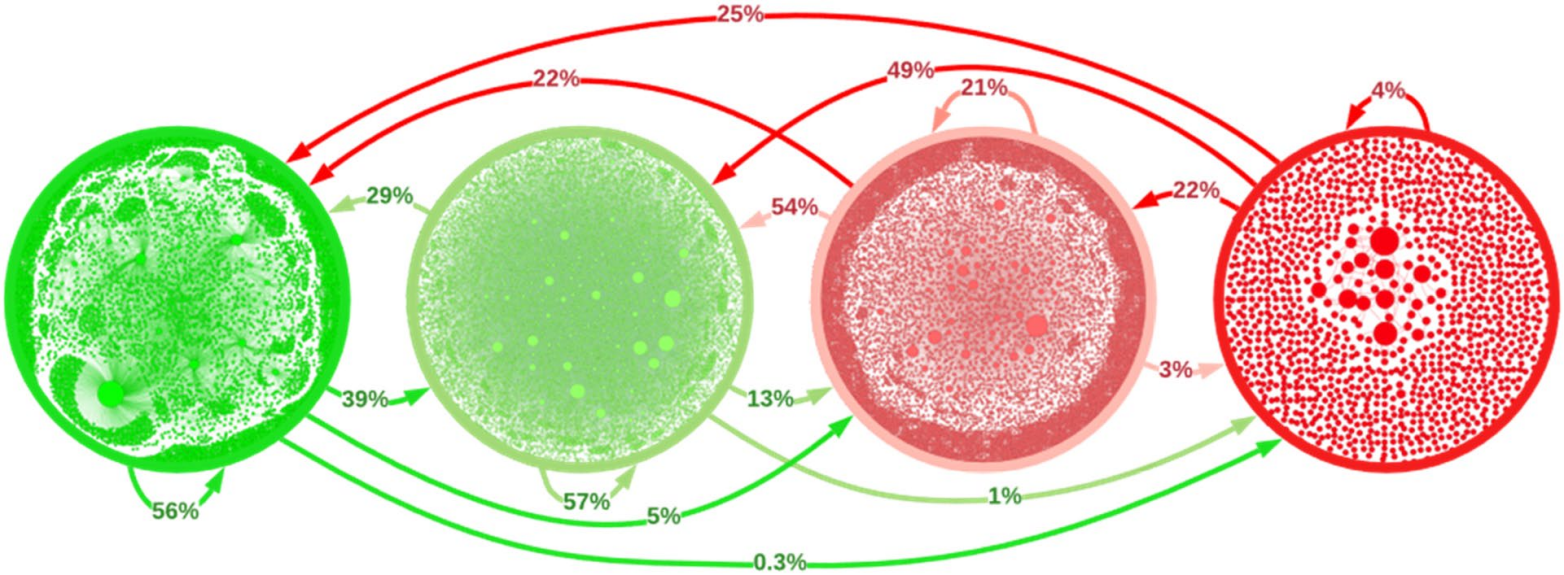
Subnetworks of only pro-vaccine (left), promoter (center-left), inhibitor (center-right), or anti-vaccine (right) nodes and their interaction flows. Each circle contains an image of the network that is obtained when considering only nodes of a given category and the links that connect them. We observe in these network images that pro-vaccine accounts form a well-connected network with a large giant component, whereas anti-vaccine accounts fragment into multiple individual nodes and small networks. Promoters and inhibitors form the largest networks, but the latter has a much lower connection density than the former. The arrows between networks show the percentage of interactions (replies, citations, and references) that accounts in each category dedicate to others. We note that pro-vaccine accounts tend to interact with other pro-vaccine accounts or with promoters, whereas anti-vaccine accounts dedicate significant efforts to interacting with pro-vaccine accounts. This can be assessed as a communications problem, since vaccine skeptics rarely have their doubts addressed by the pro-vaccine community while anti-vaccine activists intervene in most pro-vaccine conversations.

To further explore the communication between and within categories, we present in Fig. 6 the segregated networks (formed by the nodes of a single category) and the online information fluxes between them. We find that the networks for different categories display very different features, which is apparent in the diagrams displayed inside each circle. The pro-vaccine subnetwork has the highest density of internal connections (0.015% of all possible connections) and a large giant component, with over 51% of nodes. The promoter subnetwork has the second-highest connectivity density (0.0083%) and the largest giant component, with over 64% of all its nodes. The inhibitor subnetwork has the lowest internal connectivity density (0.0048%) and its giant component includes only 28% of its nodes. Finally, the anti-vaccine accounts disaggregate into multiple parts and, although the mean density of connections is not as low (0.0076%), it has a very small giant connected component, conformed by only 5% of the accounts in this category.

Figure 6 also displays the percentage of interactions (sum of all replies, citations, and references) that each category addresses to members of other categories and of its own. The results show that users that disfavor vaccination address much more often groups that favor vaccination than vice-versa. For example, over 25% of the tweets by anti-vaccine users address pro-vaccine accounts, whereas only 0.3% of pro-vaccine tweets address anti-vaccine accounts. Also, 54% of inhibitor messages address promoters, but only 13% of promoter tweets address inhibitors. Furthermore, users that favor vaccination tend to communicate within their own category, whereas those that disfavor vaccinations mainly focus on engaging with other categories. Although all this is partly due to the different number of accounts in each category, these percentages still reflect the types of accounts that their users seek to interact with. Moreover, our data show that, even after correcting for category size, pro-vaccine accounts still have a strong tendency to address likeminded accounts, whereas anti-vaccine accounts are constantly trying to argue with accounts that favor vaccination.

## Discussion and public policy implications

In sum, we find that four account categories naturally emerge from our machine learning analysis of the language used in Twitter messages. These not only distinguish between accounts that favor or disfavor vaccination but also between moderates and activists with more extreme positions on either side. We highlight the role of the newly introduced category of inhibitors, which, without necessarily being anti-vaccination, generate messages that can hinder vaccine acceptance in society, as we verified in our review of their tweets. In the Chilean case, accounts classified as inhibitors were often critical of the government or its policies through messages that could indirectly inhibit public trust in the vaccination campaign. For example, they criticized the effectiveness of the vaccine brands that had been secured by the administration, when compared to brands available in other countries. Given their larger number, these types of inhibitor positions can hinder the immunization process even more than the anti-vaccine community.

Many of our results reflect the high level of vaccine acceptance in Chile. Indeed, the low betweenness centrality of anti-vaccine and inhibitor accounts reflects their peripheral role in the Chilean conversations on vaccination. Also, despite the large number of inhibitors, we find that their opinions are near the center of the pro/anti-vaccine score range. We connect these results to the fact that, despite going through several sociopolitical conflicts, all the central articulators of the social discourse took strong pro-vaccination stances (see supplementary text for further context). Indeed, all major political parties, institutions, and public figures emphasized, above all, the need to reach high levels of immunization in Chile^9^. It would be interesting to compare our results to equivalent analyses in other countries where major political movements have taken inhibitor positions, such as focusing their discourse on the freedom to refuse vaccination.

In addition to characterizing the Chilean case, we view some of the collective behaviors found in our study as applicable to other societies. For example, the fact that the mean opinion and behavior of different account categories do not significantly change over time, while the fraction of accounts that are tweeting does reflect the state of public opinion, appears to be a behavioral property that does not depend on the country. Broadly speaking, this corresponds to stating that people tend not to change their core opinions in response to current events^32,33^, but instead become more or less motivated to express them^34^, which is consistent with the normative conformity bias^35,36^.

Even in a country with a successful vaccination process, like Chile, our analysis of online conversations reveals potential systemic issues in the pro-vaccine communication campaigns. Most notably, the fact that anti-vaccine accounts are constantly trying to argue with pro-vaccine accounts, while these mainly interact with likeminded views, reflects the dismissive attitudes that we have observed in many pro-vaccine authorities and institutions towards the anti-vaccine movement. Indeed, we noted that very few of the reviewed tweets take the time to confront anti-vaccine claims through direct argumentation. We find that these attitudes only exacerbate the movement’s conspiratorial views, further motivating their activism against the immunization process, and can leave the broader audience with no clear arguments against anti-vaccine positions.

The results of our study also suggest approaches for developing a successful digital communication strategy. These include the need to establish direct conversations with communities that disfavor vaccination. This is especially true in the case of inhibitors, since many in this category actually support vaccination and may not be clearly aware that their posts could discourage people from getting vaccinated. We believe that these users could thus be willing to reduce their inhibiting activity when this is brought to their attention. Additionally, our analyses imply that one of the main goals of online communication efforts should be to keep anti-vaccine accounts as peripheral as possible in the conversation network, as evaluated by the betweenness centrality metrics presented above. This suggests that pro-vaccine campaigns should counter highly motivated anti-vaccine activists to reduce their influence while taking care not to bring them to a more central position through these interactions, which can be achieved by engaging them from non-central accounts.

Our work demonstrates the potential use of social network data for understanding, evaluating, and managing the discussions in the digital public square regarding the Covid-19 vaccination process and other matters of public interest.

## Methods

### Data gathering

We collected all tweets written in Spanish by users in Chile between March 1st and August 31st, 2021, that contained keywords or hashtag related to the vaccines or vaccination process. We used the Twitter search API, only including accounts with public profiles. The geographical origin of each account was initially determined by the location declared in its user profile, which we compared to an extensive list of names associated to places, cities, and regions in Chile. We then filtered out all accounts from locations that have the same names but are found in other Spanish speaking countries by excluding users that employed expressions not commonly used in Chile, but typical of Spain or other Latin-American countries.

We used the following specific list of keywords and hashtags for our collection: *vacunas*, *vacuna*, *vacúnense*, *vacunado*, *vacunada*, *vacunación*, *dosis*, *Pfizer*, *Sinovac*, *CoronaVac*, *CanSino*, *AstraZeneca*, *Johnson & Johnson*, *efectos secundarios*, *coágulos*, *OMS*, *#YoMeVacuno*, and *#YoNoMevacuno.* These can be correspondingly translated to English as: *vaccines*, *vaccine*, *get vaccinated*, *vaccinated* (masculine form), *vaccinated* (feminine form), *vaccination*, *dosage*, *Pfizer*, *Sinovac*, *CoronaVac*, *CanSino*, *AstraZeneca*, *Johnson & Johnson*, *side effects*, *blood clots*, *WHO*, *#IGetVaccinated*, and *#IDontGetVaccinated*. We also included multiple variations of these terms, such as common misspellings and different forms of capitalization, accentuation, abbreviation, etc.

### Classification methodology

In order to generate data to train the machine learning algorithm, we manually classified 185 accounts. These were randomly selected from all accounts that generated between 10 and 500 tweets during the 6 months of data collection, excluding accounts from any major governmental, nongovernmental, or professional organization, as well as those that did not express a clear position or appeared to be neutral. We also verified that the selected accounts did not significantly change the positions expressed in their tweets generated throughout the 6 months of data collection, as the pandemic evolved and the vaccination process advanced.

To classify each account, we read all its collected tweets and identified the statements expressing its most pro-vaccine or most anti-vaccine views as representing its position. The view presented by each statement was evaluated based on its expected effect on its readers in favoring or disfavoring vaccination. Note that, with this approach, accounts that expressed skepticism regarding the level of protection provided by a given vaccine type or the effectiveness of the vaccination process were considered as disfavoring vaccination, despite not presenting openly anti-vaccine views.

**Figure 7:**
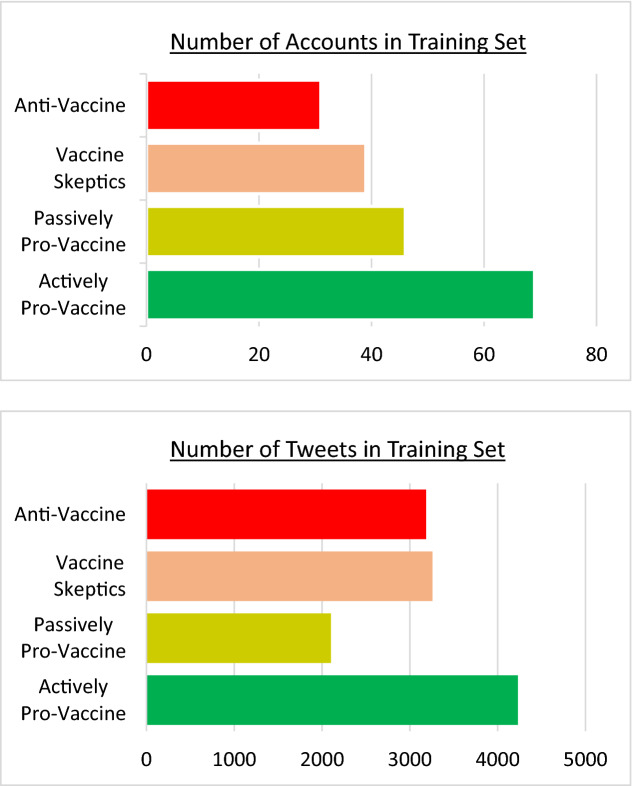
Number of accounts per category (top) and corresponding number of tweets per category (bottom) used to train our machine learning algorithm. We selected more accounts favoring vaccination than disfavoring vaccination because the former tweets less often than the latter. As a result, the total number of tweets that favor vaccination (Actively or Passively Pro-Vaccine) in the training set is similar to the total number of tweets that disfavor vaccination (Anti-Vaccine or Vaccine Skeptics) in the training set.

Following the procedure outlined above, we classified the selected accounts into four broad categories, thus identifying: 69 actively pro-vaccine accounts (explicitly encouraging vaccination), 46 passively pro-vaccine accounts (implicitly encouraging vaccination), 39 vaccine skeptic accounts (doubting the effectiveness of the vaccines or the vaccination process, or expressing honest concerns regarding specific or general side-effects), and 31 anti-vaccine accounts (explicitly discouraging vaccination or promoting fear of side-effects, often based on conspiracy theories). The accounts in these categories produced 4231, 2100, 3257, and 3185 tweets, respectively. The collection of all the texts in these tweets provided a relatively balanced, representative sample of the type of language used by the different positions and was thus well suited to be used as a training set (see Fig. 7).

Finally, we note that, in order to validate our classification method, 43 of the training set accounts were independently classified by two different individuals. We found that only two of these were not placed in the same category by the two classifiers, thus showing the consistency of our approach.

### Natural language processing steps

Before feeding the training set tweets to the model or automatically classifying all tweets, we preprocessed their texts by carrying out the following steps:

- Correction of typos and spelling errors (using the tidytext library).
- Tokenization of the text (separation of each tweet into its component words and elimination of common filler words and excess characters such as punctuation marks or emojis).
- Lemmatization (reduction of the different inflicted forms of each word into its canonical form or lemma, using the udpipe library).
- Extraction of principal lemmas (selection of only nouns, verbs, and adjectives, which contain the main meaning of a tweet, using the udpipe library).

As a result of this process, we obtained a standardized set of simplified tweets that were our starting point for all machine-based analyses.

### Machine learning implementation

In order to train the machine learning model, we binarized the four positions detailed in our Classification Methodology above by grouping them into only two categories: one favoring vaccination (combining actively and passively anti-vaccine accounts), containing 115 accounts, and one disfavoring vaccination (combining skeptic and anti-vaccine accounts), containing 70 accounts. These accounts produced a total of 6331 tweets with language favoring vaccination and 6442 tweets with language disfavoring vaccination during the 6 months of data collection, which we associated to a score of either 0 or 1, respectively, in our training process. Note that the differentiation between passively or actively pro-vaccine accounts and between skeptic or anti-vaccine accounts was lost in this step, since opinions were considered to be only binary for training purposes.

Using all these tweets by classified users, we trained a neural network model implemented with the Keras library in R and other algorithms on TensorFlow46, developed by the Google Brain team^25^. We trained with 80% of the training-set tweets and tested with the remaining 20%, using 40 epochs with batches of 512 vectors per epoch. We used the Adaptive Moment Estimation (Adam) optimizer. The network was thus trained to recognize language used by the different positions regarding vaccination. The resulting network displayed an 85% accuracy in its ability to correctly label users as favoring or disfavoring vaccination, when compared to their manual classification.

### Automatic classification

After the training process, we used the resulting neural network to classify all 205,824 tweets collected, giving each one a score between 0 and 1. Scores close to 0 correspond to tweets strongly favoring vaccination and scores close to 1, to tweets strongly disfavoring vaccination. The scores of all tweets generated by an account in the analysis period were then averaged to define a pro/anti-vaccine score for that account.

### Emerging categories

As detailed in the main text, the mean score (as computed by our trained machine learning model) of all the tweets generated by each account resulted in a natural classification of all accounts into four categories: pro-vaccine (with scores in the range 0.0–0.17), promoter (0.17–0.45), inhibitor (0.45–0.79), and anti-vaccine accounts (0.79–1.0). In order to understand what these score ranges represent, we analyzed their corresponding attitudes regarding vaccines and the vaccination process in Chile.

We provide below a brief summary of our characterization of the types of messages and accounts associated to each category.

- *Pro-vaccine accounts*: These accounts mainly used positive and informative language about the vaccines and vaccination process. They often produced messages that strongly promote vaccination. We find in this category various accounts linked to the media, the government, or municipalities, as well as accounts of ordinary users that promote the benefits of vaccination or actively call on people to get vaccinated.
- *Promoter accounts*: These accounts mostly supported the vaccines and vaccination process, although they did not strongly promote them or may have done it in a contentious way. More specifically, they typically (a) criticized health authorities and the efficacy of the vaccination process or (b) used non-empathic language, such as sarcasm or irony, when arguing against vaccine skeptics. We find in this category various members of medical associations, scientists active in social media, and pro-vaccine influencers. Some of these could approach an inhibitor score when they disseminated content that questioned the safety or effectiveness of certain vaccine brands.
- *Inhibitor accounts*: These accounts often spread messages that can reduce the readers’ willingness to get vaccinated, although they may not oppose vaccination themselves. Their tweets showed a tendency to (a) emphasize the side effects, from mild to serious, promoting any news related to them; (b) highlight and criticize any inefficiency, delay, or disorder in the vaccination centers; (c) question the effectiveness of the Chilean vaccination process; or (d) associate negative views of the government to a negative view of their vaccination policy and implementation. We find in this category a variety of users, ranging from those who present themselves as being in favor of vaccines but continuously spread news regarding potential negative side effects, to those who directly question the effectiveness of the vaccination process.
- *Anti-vaccine accounts*: These accounts used negative language that questioned the benefits of vaccination or the legitimacy of the vaccination policies, sometimes even ascribing intentionally harmful effects to vaccines. In their tweets, they typically (a) stated that they are not willing to get vaccinated, (b) promoted anti-vaccine communities, (c) disseminated content that criticized vaccines for supposedly damaging our health, and (d) presented vaccination as a threat to individual freedoms or national sovereignty. We find in this category accounts belonging to populist politicians with anti-elite platforms. Although they typically do not declare being anti-vaccine, they focus on promoting the freedom not to be vaccinated and are often connected to groups that propagate conspiracy theories that relate the pandemic and vaccines to methods supposedly developed by the elites to control society. In addition, among the most extreme anti-vaccines we also find users that believe that the purpose of vaccination is to intentionally harm the population and other conspiracy theorists of all kinds. We emphasize that our method correctly categorized, with equivalent scores, radical anti-vaccine accounts belonging to opposite extremes of the ideological spectrum.

### Characterization of pro/anti-vaccine score distribution maxima

Figure 1 (bottom panel) shows two local maxima in the account score distribution, at high and low score values, indicating that users with these scores are overrepresented in the data. By examining the distribution of scores associated to the tweets (blue line), rather than to the accounts, we find that these maxima correspond to sharp peaks where many tweets obtain very similar score values. As explained in the main text, the reason for these peaks is that the more extreme pro- and anti-vaccine views tend to use repetitive language with monolithic messages that actively promote their positions. In order to demonstrate this point, we discuss below our manual inspection of the language associated to the most highly overrepresented scores, labeled by red dashed vertical lines in Fig. 1.

- **Peak at score = 0.055:** Our manual inspection of tweets near this score shows that they correspond to messages that promote official information on the vaccination calendar established by Chilean authorities. They typically contained information, comments, or questions (often generated by accounts of local governments or authorities) regarding the practicalities of the vaccination process, in relation to issues such as the location of vaccination centers or progress in the vaccination schedule. More specifically, many of the tweets included the following terms or word combinations: *vaccination process*, *vaccinate*, *vaccinated*, *schedule*, *get*, *dose*, *booster*, and *#IGetVaccinated* (corresponding in Spanish to: *proceso vacunación*, *vacunar*, *vacunados*, *calendario*, *recibir*, *dosis*, *refuerzo*, and *#YoMeVacuno*). In particular, we find many tweets that contribute to this peak with the same identical score (0.05666366), which post almost the same message when announcing where and when certain age groups should get vaccinated.
- **Peak at score = 0.125:** The tweets near this score tend to correspond to conversations regarding the vaccination with specific brands available in Chile, especially AstraZeneca and Sinovac, the most commonly used vaccines in the country at the time. Many of the tweets included the following terms or word combinations: *first dose*, *second dose*, *vaccinate*, *adverse*, *booster*, *effectiveness*, *symptoms*, *mobility*, and *pass* (corresponding in Spanish to: *primera dosis*, *segunda dosis*, *vacunar*, *adverso*, *refuerzo*, *efectividad*, *síntoma*, *movilidad*, and *pase*). In particular, we find many tweets with the same identical score (0.1252309), which discuss the potential benefits of a particular vaccine brand.
- **Peak at score = 0.835:** The tweets near this score tend to use similar stereotypical language opposing vaccination or specific brands (typically AstraZeneca and Sinovac). We find near this score various tweets from different accounts that promote common anti-vaccine messages with terms such as *side-effects*, *kill*, *death*, *experimental*, etc. (corresponding in Spanish to: *efectos secundarios*, *mata*, *muerte*, *experimental*, etc.) In particular, we find many posts with identical score (0.8380035), corresponding to very short messages by a diversity of individual accounts that included the hashtag “#IDon’tGetVaccinated” (in Spanish: “#YoNoMeVacuno”).

## Supplementary Information

Supplementary Information 1.

Supplementary Information 2.

Supplementary Information 3.

## Data Availability

All data are available in the main text or the supplementary materials.

## Code availability

All the codes used for data processing are available upon request.
