# Supplementary material for: Influence of online opinions and interactions on the Covid-19 vaccination in Chile
Source: Sci Rep. 2022 Dec 9;12:21288. doi: 10.1038/s41598-022-23738-0 (PMC9734170; doi:10.1038/s41598-022-23738-0)
Supplement: Supplementary file 3 — Supplementary Information 3. [file 41598_2022_23738_MOESM3_ESM.pdf]

Supplementary Text:

"Influence of online opinions and interactions on the Covid-19 vaccination in Chile"

Authors: Claudio Villegas, Abril Ortiz, Víctor Arriagada, Sofía Ortega, Juan Walker, Eduardo Arriagada, Alexis M. Kalergis*, Cristián Huepe*

Supplementary Text: Context of the Chilean Vaccination Process

To contextualize the complex sociopolitical dynamics surrounding the Chilean vaccination campaign and the related online conversations analyzed in this paper, we overview here some of the main events that occurred in the country before and during our period of study. In general terms, the discussions on the vaccination process were strongly affected in Chile by two recent sociopolitical issues: a major "social outburst" that developed at the end of 2019 and the irregular performance of the government during the first year of the pandemic. We will describe events related to both of these, more in detail, below.

As in almost all the rest of the world, the Chilean vaccination process was greatly hindered by the misinformation that abounds in online social networks[1,32,33]. In Chile, as in most countries with high levels of vaccine access, a large majority of the population gets their news from these same social networks[4]. Indeed, over 92% of the Chilean population uses the internet[3] and over 80% of the respondents to recent polls declared getting their news daily from online social networks (Facebook, Instagram, Twitter, etc.) while another 13% does so every few days. On the other hand, these high levels of access to information are accompanied by a deep mistrust in traditional journalism. Indeed, 89% of respondents believed that journalists do not cover important facts and 85%, that they do not interview the right people[34].

Online news and conversations regarding the vaccines and vaccination campaign in Chile were strongly affected by the fact that, just five months before the media started talking about Covid, Chile had experienced the biggest social insurrection of its last 30 years. On October 18, 2019, this "social outburst" began as a combination of massive peaceful demonstrations with social unrest and violent clashes that led to 20 fatalities and thousands of injured, both in the civilian population and among the police[10]. A first night of violent crashes ended with the burning of an electricity company building and seven subway stations in the city. Violence escalated in the following days, spreading throughout the country. Seventy more stations were damaged; hundreds of supermarkets and pharmacies were looted[10-12]. This social outburst was caused by a generalized feeling that the economic differences in Chilean society had become intolerable due to repeated abuses and price increases in basic services, transportation, and health, as well as the low pensions[10,12]. This led to a strong distrust in institutions, political parties, and elites, similar to that experienced in many other countries[11,12].

The social outburst was organized through an intensive use of online social networks[34]. The protesters took advantage of the almost universal access to smartphones achieved in Chile by 2019, which allowed them to stay always connected[10]. Indeed, polling showed that 86% of respondents acknowledged using smartphones to coordinate protests, which is similar to the percentages that stated using online social media to obtain their news and that believed that traditional media did not do well their job[12].

As the violence linked to the social outburst grew, the president declared in an address to the nation that "we are at war against a powerful, implacable enemy", when referring to violent protesters, and the government declared martial law and established a curfew[10]. However, the protests gained support among large segments of the population and, only a week after, over a million Chilean demonstrators organized what was referred to as the largest "peaceful and festive" demonstration in the history of the country[11]. As the crisis continued to develop, about a month after the beginning of the social outburst, although many protests remained peaceful, violent clashes also increased to the point that almost all government and opposition political parties signed an agreement for a new social pact on November 15, 2019, which started a process to write a new Constitution[11,12]. In January and February 2020, with the arrival of summer and of the period of vacations, violence had decreased, although sporadic confrontations between the police and demonstrators continued[11].

The pandemic response further polarized the society when Covid-19 arrived in Chile, since the government reinstated a state of exception and curfew to limit viral transmission (which had been used 5 months earlier to control the social outburst) just as the mass protests were expected to return[11,12]. This linked the Chilean population's view of the pandemic with their political attitudes, as it also occurred in many other countries. In this context, various NGOs, think tanks, professional medical associations and the academic community became strongly involved in the public discussions regarding the government's pandemic mitigation efforts, in agreement with or in opposition to the presidential administration[35].

The controversies surrounding the pandemic response were also driven by the difficulties experienced by the government in controlling the spread of the virus. In June 2020, as a significant portion of the population was getting vaccinated, Chile reached one of the highest transmissibility rates in the world and its highest mortality rates of the pandemic[4], as shown in Fig. S1. This happened even after drastic measures that curtailed individual freedoms had been imposed, such as declaring a state of "sanitary emergency", a curfew from 10 p.m. to 6 a.m., and strict quarantines in communities with high contagion rates. Although it initially slowed down the spread of the disease, this strategy soon failed to contain infections due to the lack of sufficient economic assistance for the most vulnerable, which had to leave their homes to survive[2]. As in other countries, there were also many initial failures in the public communication strategy and the implementation of containment measured. Authorities were criticized for emitting contradictory messages, for generating a false sense of security by imposing dynamic quarantines that closed whole neighborhoods without effectively limiting travel to and from

them, and for their initial delay in taking advantage of a well-established public primary health network[5].

Despite the significant sociopolitical debate surrounding the Chilean pandemic response described above (which, we note, mainly developed on social networks due to the imposed mobility restrictions), the government and the academic and scientific communities rapidly aligned behind efforts to procure vaccines early and to distribute them efficiently to its population. Chile thus became one of the first countries that carried out clinical trials for the Covid-19 vaccines that were being developed by various laboratories around the world[6,19,20]. It also rapidly secured vaccines for its whole population and established an efficient public vaccination schedule. In addition, while they expressed different positions regarding their support or opposition to the government, all major opinion leaders, organizations, and media outlets strongly supported the vaccination campaign. This has led Chile to achieve one of the highest immunization levels in the world, although there are still over 1.2 million in the target population that have not yet received a single dose or completed their vaccination schedule, as of January 7, 2022[6].

Additional Supplementary Text References

32. F. M. Magarini, M. Pinelli, A. Sinisi, S. Ferrari, G. L. De Fazio, G. M. Galeazzi, Irrational beliefs about COVID-19: A scoping review. *Int. J. Environ. Res. Public Health.* **18**(19), 9839 (2021).
[doi: 10.3390/ijerph18199839. PMID: 34639241; PMCID: PMC8508358].

33. G. M. Nieves-Cuervo, E. F. Manrique-Hernández, A. F. Robledo-Colonia, A. E. K. Grillo. Infodemia: noticias falsas y tendencias de mortalidad por COVID-19 en seis países de América Latina. *Rev. Panam. Salud Publica*. 45:e44 (2021).
[https://doi.org/10.26633/RPSP.2021.44]

34. S. F Tsao, H. Chen, T. Tisseverasinghe, Y. Yang, L. Li, Z. A. Butt, What social media told us in the time of COVID-19: a scoping review. *Lancet Digit Health*. Mar, **3** (3) (2021):e175-e194.
[doi: 10.1016/S2589-7500(20)30315-0. PMID: 33518503; PMCID: PMC7906737].

35. I. Bachmann, S. Valenzuela, A. Figueroa-Bustos, COVID-19 in Chile: A health crisis amidst a political crisis amidst a social crisis, in *Political communication in the time of Coronavirus* (Routledge, 2021). pp. 48–64.

Fig. S1.

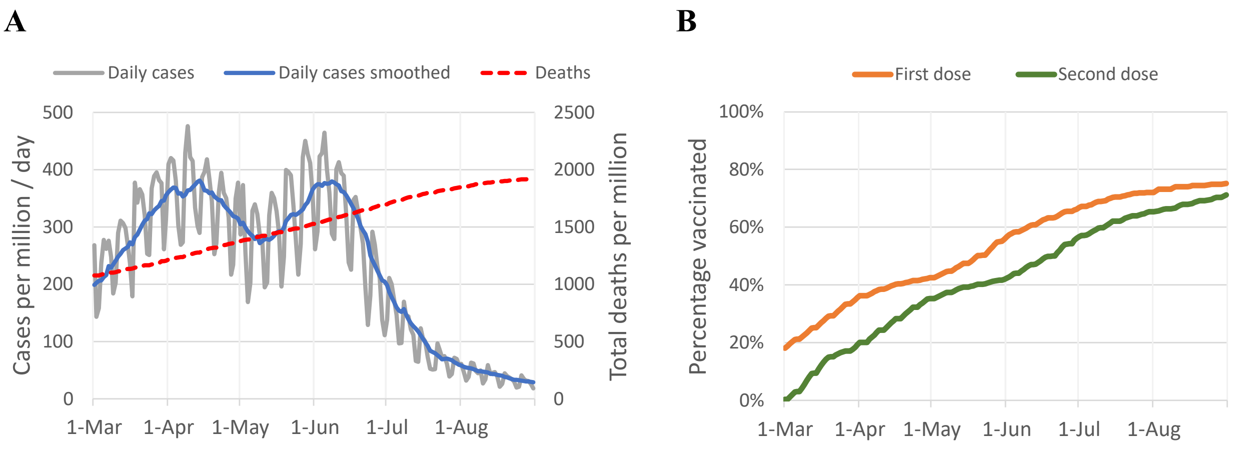

Dynamics of the Covid-19 pandemic in Chile *(4)* during the 6 moths analyzed in this study, from March 1st till August 31st, 2021. (A) Number of cases per million people per day (solid curves, left-side axis) and total deaths per million (dashed line, right-side axis). **(B)** Total fraction of the population that received their first and second dose. We observe a strong variation in the number of cases, which increased and decreased twice between Mach and June. During July and August, they decayed to significantly lower levels. At the same time, the number of deaths rose steadily, almost doubling during the analyzed period, while the immunized fraction of the populations increased from almost nothing to close to 80%. The vaccination program was most controversial when high infection levels, deaths and vaccination rates were simultaneously observed (mainly the beginning of June 2021).

Supplementary Table - data S1

Classified Training Set Accounts

Table of all manually classified accounts used as the training set for our machine learning model. The first column contains the username of each account; the second column, its corresponding manual classification. Actively and passively pro-vaccine accounts are given training scores equal to zero, whereas vaccine skeptic and anti-vaccine accounts are given training scores equal to one. Note that we also include here accounts classified as "Neutral", for completeness, although they were not used in the training process.

Supplementary Table - data S1

Analyzed Tweets with Scores

Table of all tweets analyzed in this work. The first column contains the URL link to each original tweet; the second column, the lemmatized version of the tweet's content; the third, the username of the account that produced the tweet; and the fourth, the tweet's pro/anti-vaccine score, as computed by our machine learning model trained with the tweets in Data S1.
